# The influence of family care guidance for premature infants based on a telemedicine model on their neurodevelopmental assessment indicators

**DOI:** 10.3389/fped.2026.1636574

**Published:** 2026-04-23

**Authors:** Li Zuo, Huichu Ye, Daoxing Li

**Affiliations:** Department of Pediatrics, Beijing Friendship Hospital Affiliated to Capital Medical University, Beijing, China

**Keywords:** family care guidance, home care, neurodevelopmental assessment, premature infants, randomized controlled trial, telemedicine

## Abstract

**Background:**

Premature infants, defined as those born before 37 weeks of gestation, are at elevated risk for neurodevelopmental delay due to incomplete organ maturation and heightened vulnerability to neurological impairment during the critical early postnatal period. Family care plays a crucial role during their growth process, yet the traditional nursing model has substantial temporal and spatial limitations. Telemedicine models offer a promising new solution for the family care of premature infants; however, existing research examining the impact of telemedicine-based interventions on neurodevelopmental outcomes has notable deficiencies, including limited sample sizes, short follow-up periods, and inconsistent outcome measures, leaving the overall effectiveness of such interventions not yet fully established.

**Methods:**

This single-center randomized controlled trial enrolled 186 premature infants admitted to the neonatal department of Beijing Friendship Hospital from January 2023 to December 2024. Infants were randomly assigned to an observation group (*n* = 93) or a control group (*n* = 93) using a computer-generated random number table. Sample size was determined by *a priori* power analysis (*α* = 0.05, power = 0.80) based on expected effect sizes from prior telemedicine intervention studies. The control group received routine home care according to standard hospital practice, while the observation group received additional home care guidance based on a telemedicine model, including monthly 60-minute remote video training sessions, 24/7 real-time online consultation, and weekly personalized knowledge push via WeChat. The intervention period was 6 months, beginning at hospital discharge. Blinded assessors evaluated outcomes using the Bayley Scales of Infant and Toddler Development (Third Edition), Neonatal Behavioral Neurological Assessment, Peabody Developmental Motor Scales (Second Edition), Social Adaptive Behavior Scale (infant subscales), and Neuropsychological Development Scale for Children aged 0–6 years. Daily behavioral observation indicators including sleep regularity, feeding quality, and emotional stability were recorded by parents using standardized definitions provided during training.

**Results:**

After the 6-month intervention, the observation group demonstrated significantly higher scores across all domains of the Bayley Scales (cognitive: 87.6 ± 4.9 vs. 81.3 ± 4.5, Cohen's d = 1.34; language: 85.2 ± 4.6 vs. 79.1 ± 4.2, Cohen's d = 1.39; motor: 86.3 ± 4.7 vs. 80.5 ± 4.3, Cohen's d = 1.29; all *P* < 0.001). The NBNA score (37.8 ± 1.4 vs. 35.6 ± 1.2, Cohen's d = 1.69, *P* < 0.001), gross motor function score (88.4 ± 5.8 vs. 81.7 ± 5.0, Cohen's d = 1.24, *P* < 0.001), fine motor function score (87.7 ± 5.6 vs. 80.3 ± 4.8, Cohen's d = 1.42, *P* < 0.001), Social Adaptability Scale score (86.8 ± 5.3 vs. 82.6 ± 4.9, Cohen's d = 0.82, *P* < 0.001), and Neuropsychological Development Questionnaire score (85.7 ± 5.4 vs. 79.8 ± 4.8, Cohen's d = 1.15, *P* < 0.001) were all significantly higher in the observation group compared with the control group. Among daily behavior observation indicators, the rates of meeting standards for sleep regularity (82.8% vs. 67.7%, *χ*^2^ = 7.917, *P* = 0.005), good feeding (79.6% vs. 64.5%, *χ*^2^ = 6.356, *P* = 0.012), and emotional stability (86.0% vs. 72.0%, *χ*^2^ = 7.021, *P* = 0.008) were significantly higher in the observation group.

**Conclusion:**

Family care guidance for premature infants based on a telemedicine model can effectively improve multiple neurodevelopmental assessment indicators and daily behavioral outcomes. This intervention addresses previous research limitations by employing rigorous randomization, standardized outcome measures, and adequate sample size, thereby providing robust evidence for an efficient and convenient new approach for family care of premature infants that is worthy of clinical promotion and application.

## 1 Introduction

The term premature infants refers to those whose gestational age at birth is less than 37 weeks (1). In recent years, with the rapid development of perinatal medicine, the survival rate of premature infants has significantly increased. However, due to the immature development of their various organ systems, they are prone to various complications after birth, especially abnormal development of the nervous system, which seriously affects their long-term quality of life (2, 3). According to statistics, the global incidence of premature birth is approximately 10% and shows an increasing trend year by year (4). In China, the birth rate of premature infants is also as high as 7% to 8%, with approximately 1.1 million premature infants born each year (5). Neurodevelopmental delay in premature infants not only brings a heavy economic burden and mental stress to families but also poses a significant challenge to social medical resources. Therefore, how to effectively promote the neurodevelopment of premature infants has become a research hotspot in the fields of perinatal medicine and child health care.

The development of the nervous system is highly active during the early postnatal period of premature infants and is susceptible to various environmental and biological factors. Extensive research has demonstrated that, due to the immature brain development of premature infants, the brain development process that is not completed *in utero* needs to continue in the extrauterine environment, which makes them face many risks during the process of neural development (6, 7). The incidence of white matter injury in premature infants is relatively high, which may lead to long-term cognitive, motor, and behavioral disorders (8). Meanwhile, the establishment and improvement of neural reflexes in premature infants may also be delayed; for instance, primitive reflexes such as the sucking reflex and grasping reflex may weaken or disappear at delayed intervals, and physiological reflexes such as the knee-jerk reflex and Achilles tendon reflex may occur at abnormal times, all of which reflect the immaturity of their nervous system development (9). Electroencephalogram monitoring often shows abnormal brain electrical activity in premature infants, suggesting potential risks in their brain physiological functions and pathological changes (10). Imaging examinations such as ultrasound and MRI can directly observe the brain structure and functional development of premature infants, discover problems such as abnormal brain structure and developmental disorders, and further confirm the fragility of the nervous system development of premature infants (11).

As the primary environment for the growth of premature infants, the quality of care in the family plays a key role in their neural development. Family care encompasses multiple aspects that are interrelated and jointly influence the neurodevelopment process of premature infants. Reasonable nutritional supply is the material basis for the growth and development of premature infants; the use of breast milk fortifiers can effectively supplement the deficiency of nutrients in the breast milk of premature infants and promote their physical and brain development (12). In terms of daily care, creating a quiet and comfortable environment, reducing light and sound stimulation, and adopting developmental care methods such as bird's nest care and kangaroo care can simulate the uterine environment and have a positive impact on the neurobehavioral development of premature infants (13). Kangaroo mother care has been shown to improve long-term breastfeeding rates and neurodevelopment in preterm infants (10). Meanwhile, parents’ mastery and application of disease prevention knowledge for premature infants, such as timely detection and handling of problems like infections, can reduce the adverse effects of diseases on neural development (14). However, the traditional home care model has many limitations. Due to the lack of professional medical knowledge and nursing skills, parents often feel overwhelmed when facing the complex nursing needs of premature infants. In terms of feeding, it is difficult to accurately grasp the feeding amount and frequency of premature infants, resulting in insufficient nutrient intake or overfeeding. Regarding disease observation, parents without specialized training may have difficulty identifying abnormal symptoms in a timely manner, potentially delaying treatment opportunities (15). Survey data from our institution and prior literature indicate that geographic distance from tertiary medical centers and transportation barriers create substantial obstacles for regular in-person follow-up visits, which affects the timely monitoring and intervention of the neurodevelopment of premature infants (16, 17).

With the rapid development of information technology, telemedicine models have emerged and have gradually been applied in the field of medical care. Telemedicine utilizes means such as the Internet and communication technologies to break through the limitations of time and space and achieve optimal allocation of medical resources (18). In the home care of premature infants, telemedicine models have significant advantages. Through remote video training, medical staff can visually demonstrate to parents the care techniques and key operational methods for premature infant care, such as the correct feeding posture and umbilical cord care, thereby improving parents’ care skills (19). The real-time online consultation function enables parents to receive professional answers promptly when encountering problems, enhancing their confidence in care (20). Personalized knowledge push, defined as the targeted delivery of educational materials and care recommendations based on individual infant characteristics (such as gestational age, birth weight, and medical history) and parental learning needs through digital platforms, can provide parents with targeted care suggestions and neurodevelopment promotion plans, achieving precise care (21). Although telemedicine models have brought new opportunities for home care of premature infants, current research on their impact on neurodevelopmental assessment indicators remains limited. Prior studies have shown initial positive effects of telemedicine in the care of premature infants; however, important deficiencies persist in the literature. Specifically, many studies have used small sample sizes with inadequate statistical power, have employed inconsistent or non-standardized outcome measures, have lacked proper randomization and blinding procedures, and have had short follow-up periods that preclude assessment of sustained effects (22, 23). The present study was designed to address these specific methodological limitations by employing rigorous randomization, blinded outcome assessment, validated standardized neurodevelopmental instruments, adequate sample size determined by *a priori* power analysis, and a six-month intervention period with comprehensive outcome evaluation.

## 2 Methods

### 2.1 Ethics approval

This study was reviewed and approved by the Ethics Committee of Beijing Friendship Hospital Affiliated to Capital Medical University prior to study initiation. The ethics approval number is: [2023] Lun Shen No. (079). All research was conducted in strict accordance with the Declaration of Helsinki. All family members of the premature infants provided written informed consent before enrollment.

### 2.2 Study design and participants

This single-center, parallel-group randomized controlled trial was conducted at the neonatal department of Beijing Friendship Hospital. Beijing Friendship Hospital is a tertiary medical center located in Beijing, China, serving a diverse urban and suburban population with varying socioeconomic backgrounds. All premature infants admitted to the neonatal department from January 2023 to December 2024 were systematically screened for eligibility. During this period, a total of 247 premature infants were screened, of whom 186 met eligibility criteria and were enrolled.

**Figure 1:**
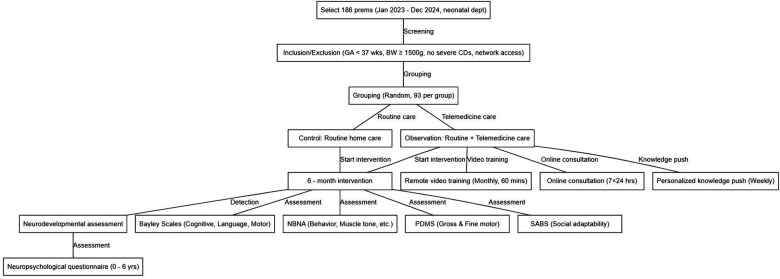
The technical roadmap of the research.

Inclusion criteria were as follows: gestational age at birth less than 37 weeks (indicating prematurity at the time of delivery); birth weight 1,500 grams or greater; absence of serious congenital diseases such as congenital heart disease requiring surgical intervention or neural tube defects; family has basic network conditions including smartphone with video capability and stable internet connection; and family can cooperate to complete the relevant operations of telemedicine. Exclusion criteria were as follows: transfer to another hospital or death due to critical condition during hospitalization; parents have cognitive impairments or are unable to cooperate with the research; and withdrawal from the research after enrollment. The participant flow diagram (Figure 1) illustrates the complete screening, enrollment, randomization, and follow-up process in accordance with CONSORT guidelines. NICU indicates neonatal intensive care unit; FU indicates follow-up; Obs indicates observation group; Ctrl indicates control group.

### 2.3 Sample size determination

Sample size was calculated *a priori* using G*Power software (version 3.1.9.7). Based on previous telemedicine intervention studies in premature infant populations reporting effect sizes of Cohen's d ranging from 0.50 to 0.80 for neurodevelopmental outcomes (22, 24), we assumed a medium-to-large effect size of d = 0.60. With *α* = 0.05 (two-tailed) and power = 0.80, the minimum required sample size was 90 participants per group. To account for potential attrition of up to 5%, we enrolled 93 participants per group, for a total of 186 participants.

### 2.4 Randomization and blinding

Randomization was performed using a computer-generated random number sequence created by an independent statistician not involved in participant enrollment or outcome assessment. The 186 enrolled infants were assigned to the observation group or the control group in a 1:1 ratio, with 93 cases in each group. The allocation sequence was concealed using sequentially numbered, opaque, sealed envelopes prepared by the statistician. Randomization was performed at the time of hospital discharge, immediately before the intervention period began. The allocation was revealed to the family and the clinical team providing the telemedicine intervention only after randomization was complete. Outcome assessors conducting neurodevelopmental evaluations at the 6-month endpoint were blinded to group assignment. Due to the nature of the intervention, participants and clinical staff delivering the intervention could not be blinded.

### 2.5 Baseline assessment

To establish baseline equivalency between groups, the following assessments were performed prior to hospital discharge in addition to demographic characteristics. Length of hospital stay was recorded (observation group: 18.3 ± 7.2 days; control group: 17.9 ± 6.8 days; *t* = 0.384, *P* = 0.701). Causes of prematurity were documented, including preterm premature rupture of membranes (observation: 28.0%; control: 25.8%), preeclampsia (observation: 18.3%; control: 21.5%), placental abnormalities (observation: 15.1%; control: 14.0%), multiple gestation (observation: 22.6%; control: 24.7%), and other/unknown causes (observation: 16.1%; control: 14.0%), with no significant differences between groups (*χ*^2^ = 1.247, *P* = 0.870). Number of medications at discharge was also recorded (observation group: 1.8 ± 0.9; control group: 1.9 ± 1.0; t = 0.694, *P* = 0.489). Baseline NBNA scores were obtained prior to discharge (observation group: 32.4 ± 2.1; control group: 32.1 ± 2.3; t = 0.897, *P* = 0.371), confirming baseline equivalency in neurobehavioral status.

### 2.6 Intervention methods

#### 2.6.1 Control group

The control group received routine home care according to the hospital's standard practice. This included face-to-face health education conducted by medical staff before discharge, covering knowledge such as feeding of premature infants (including breastfeeding methods and formula milk preparation ratios), skin care (including umbilical cord care and diaper changes), and disease observation (including symptom identification of fever, cough, and diarrhea). After discharge, outpatient follow-up was conducted according to standard protocol, with visits at 1 week and 2 weeks after discharge, and then monthly thereafter. At each follow-up visit, pediatricians conducted routine physical examinations and growth and development evaluations.

#### 2.6.2 Observation group

In addition to receiving the same routine home care as the control group, the observation group received home care guidance based on a telemedicine model. The intervention began at the time of hospital discharge and continued for 6 months. All families in the observation group used the same dedicated telemedicine platform, and parents were trained on its use during hospitalization before discharge.

##### 2.6.2.1 Remote video training

A dedicated telemedicine platform was established utilizing a secure, HIPAA-compliant video conferencing system integrated with WeChat for accessibility. Remote video training sessions of approximately 60 min were conducted monthly. The training courses were jointly taught by neonatologists, nurses, and rehabilitation therapists. The content included the neurodevelopmental characteristics of premature infants, early family intervention methods (such as touch, passive movement, and other operational skills to promote neurodevelopment), and handling of common problems (such as first aid for choking on milk and coping with apnea). During the training process, interactive sessions were incorporated, during which parents could ask questions at any time and instructors provided immediate answers.

##### 2.6.2.2 Real-time online consultation

Through the instant messaging function of the telemedicine platform, 24 h-a-day, 7-day-a-week real-time online consultation services were provided for parents. Professionally trained medical staff took turns being on duty, promptly responding to parents’ questions regarding the care, feeding, and disease observation of premature infants. For complex issues, multi-disciplinary experts were coordinated for remote consultations.

##### 2.6.2.3 Personalized knowledge push

By leveraging data analysis technology, personalized care knowledge and neurodevelopment promotion plans were pushed weekly through text messages, WeChat official accounts, or the telemedicine platform based on the individual conditions of premature infants (such as gestational age, birth weight, and disease history) and the expressed needs of parents. The content was presented in the form of pictures, text, and short videos, which was convenient for parents to understand and implement.

### 2.7 Intervention adherence

Intervention adherence was carefully monitored throughout the study period. On average, families in the observation group participated in 5.7 ± 0.5 of the 6 planned monthly video training sessions (adherence rate: 95.0%). The mean number of online consultations per family was 8.3 ± 3.2 over the 6-month period. All families received the weekly personalized knowledge push as planned. No families withdrew from the observation group after randomization. This study was unfunded; resources for implementing the telehealth care included existing hospital personnel, the institution's telemedicine infrastructure, and freely available communication platforms (WeChat). No significant barriers to establishing the telehealth intervention were encountered, as families were pre-screened for network capability and device access prior to enrollment.

### 2.8 Outcome measures and evaluation methods

Outcomes were evaluated at the end of the 6-month intervention period. The primary outcomes were scores on the Bayley Scales of Infant and Toddler Development (cognitive, language, and motor domains), as these directly relate to the study objective of assessing neurodevelopmental improvement. Secondary outcomes included NBNA score, Peabody Developmental Motor Scale scores (gross and fine motor), Social Adaptive Behavior Scale score, Neuropsychological Development Questionnaire score, and daily behavioral observation indicators. This hierarchical prioritization of outcomes guides interpretation, with the primary outcomes being the principal focus of analysis. All neurodevelopmental assessments were conducted by trained assessors who were blinded to group assignment. Assessments were performed in a standardized examination room at Beijing Friendship Hospital.

#### 2.8.1 Bayley scales of infant and toddler development, third edition (bayley-III)

The Bayley-III is a gold-standard, individually administered instrument for assessing the developmental functioning of infants and toddlers aged 1–42 months (25). The scale yields composite scores (mean = 100, SD = 15) in three domains: Cognitive, Language (combining Receptive and Expressive Communication), and Motor (combining Fine and Gross Motor). Higher scores indicate better developmental status. The Bayley-III has demonstrated excellent psychometric properties, with test-retest reliability coefficients of 0.80–0.90 and concurrent validity with other developmental measures of 0.60–0.80 (25). The Chinese adaptation has been validated for use in Chinese infant populations.

#### 2.8.2 Neonatal behavioral neurological assessment (NBNA)

The NBNA is a standardized assessment tool for evaluating the neurobehavioral status of neonates and young infants (26). The assessment measures five domains: behavioral ability, passive muscle tone, active muscle tone, primitive reflexes, and general condition. The full score is 40 points, with higher scores indicating better neurobehavioral development. The NBNA has demonstrated good reliability (inter-rater reliability: 0.85–0.92) and validity for detecting neurological abnormalities in premature infants (26).

#### 2.8.3 Peabody developmental motor scales, second edition (PDMS-2)

The PDMS-2 is a norm-referenced standardized instrument for assessing gross and fine motor skills in children from birth through 5 years of age (27). The Gross Motor Quotient assesses reflexes, stationary, locomotion, and object manipulation. The Fine Motor Quotient assesses grasping and visual-motor integration. Standard scores have a mean of 100 and SD of 15. The PDMS-2 has excellent psychometric properties, with internal consistency coefficients of 0.89–0.97 and test-retest reliability of 0.89–0.93 (27).

#### 2.8.4 Social adaptive behavior scale (SABS)

The SABS is a parent-report measure of social adaptive functioning (28). For this study, only the infant and early childhood subscales appropriate for the 6–12 month age range were administered, assessing domains relevant to this developmental period including independent living skills (feeding, sleeping), social interaction, and communication. The school-related domains and items designed for older children were not administered, as they are not applicable to infants under 12 months of age. Higher scores indicate better social adaptability. The infant subscales have demonstrated adequate reliability (Cronbach's α = 0.78–0.85) and content validity (28).

#### 2.8.5 Neuropsychological development scale for children aged 0–6 years

This is a standardized Chinese assessment instrument evaluating neuropsychological development across five dimensions: gross motor skills, fine motor skills, adaptability, language, and social behavior (29). Parents completed questionnaires based on their children's daily performance, and the comprehensive score reflected the overall level of neuropsychological development. The scale has demonstrated good reliability (test-retest reliability: 0.82–0.88) and has been validated in Chinese pediatric populations (29).

#### 2.8.6 Daily behavioral observation indicators

Parents were provided with standardized definitions and trained during the video sessions to record the following indicators. Sleep regularity was defined as stable daily sleep duration (14–17 h for infants 4–11 months, as per American Academy of Sleep Medicine recommendations) with consistent sleep-wake schedules (falling asleep and waking within 30 min of the same time each day). Good feeding was defined as adequate daily milk intake meeting age-appropriate volume requirements with no more than 2 episodes of vomiting per week. Emotional stability was defined as the absence of prolonged crying episodes (lasting more than 20 min without identifiable cause) and the infant being easily soothed when distressed. Compliance rates for each indicator were calculated at 6 months post-intervention based on parent-reported data from the final 2 weeks of the intervention period.

### 2.9 Statistical methods

Data analysis was conducted using SPSS version 26.0 statistical software. Continuous variables conforming to normal distribution are expressed as mean ± standard deviation. Between-group comparisons were conducted using independent sample *t*-tests. Categorical variables are expressed as frequencies and percentages, with between-group comparisons conducted using chi-square tests. Given the multiple outcome comparisons, the Benjamini-Hochberg procedure was applied to control the false discovery rate at 0.05 for the primary outcomes. Effect sizes were calculated as Cohen's d for continuous variables (interpreted as small: 0.2, medium: 0.5, large: 0.8) and Cramér's V for categorical variables. 95% confidence intervals for effect sizes are reported. A two-tailed *P* < 0.05 was considered statistically significant.

## 3 Results

### 3.1 Analysis of demographic and social characteristics

A total of 186 premature infants admitted from January 2023 to December 2024 were randomly assigned to the observation group (93 cases) and the control group (93 cases). No participants were lost to follow-up, resulting in complete data for all 186 enrolled infants. The 0% attrition rate can be attributed to several factors: the strong therapeutic alliance established through the telemedicine platform, the convenience of remote participation which eliminated transportation barriers, regular contact maintaining engagement, and the high motivation of parents of premature infants to optimize their child's developmental outcomes. Additionally, control group families were motivated by the regular follow-up assessments and the opportunity to contribute to research benefiting future premature infants.

The demographic data of the two groups were compared and are presented in Table 1. In terms of gestational age of premature infants (observation: 34.2 ± 1.5 weeks; control: 34.5 ± 1.3 weeks; *t* = 1.447, *P* = 0.150), birth weight (observation: 1,850.2 ± 200.3 g; control: 1,820.5 ± 190.6 g; *t* = 0.992, *P* = 0.323), parents’ age (observation: 28.5 ± 3.2 years; control: 29.0 ± 3.0 years; *t* = 1.072, *P* = 0.286), parental educational level (*χ*^2^ = 1.136, *P* = 0.769), and monthly household income (observation: 8,500 ± 1,500 yuan; control: 8,200 ± 1,600 yuan; *t* = 1.127, *P* = 0.261), there were no significant differences between groups. For context, the mean monthly household income of 8,200–8,500 yuan (approximately USD 1,140–1,180) represents a middle-income level in urban China, above the national median but below upper-income thresholds. These results indicate that the baseline data of the two groups were balanced and comparable, which effectively ensures the validity and reliability of the subsequent intervention effect analysis.

**Table 1:** Analysis of demographic and social characteristics.

Variable	Observation group (*n* = 93)	Control group (*n* = 93)	t/*χ*^2^	*P*
Gestational age (weeks)	34.2 ± 1.5	34.5 ± 1.3	1.447	0.150
Birth weight (g)	1,850.2 ± 200.3	1,820.5 ± 190.6	0.992	0.323
Parents’ age (years)	28.5 ± 3.2	29.0 ± 3.0	1.072	0.286
Educational attainment	–	–	1.136	0.769
Primary school and below	10 (10.8%)	12 (12.9%)	–	–
Junior high school	25 (26.9%)	22 (23.7%)	–	–
High school/technical school	30 (32.3%)	33 (35.5%)	–	–
Junior college and above	28 (30.1%)	26 (28.0%)	–	–
Monthly household income (yuan)	8,500 ± 1,500	8,200 ± 1,600	1.127	0.261

Continuous variables expressed as mean ± SD; categorical variables expressed as *n* (%).

### 3.2 Comparison of neurodevelopmental assessment indicators

**Figure 2:**
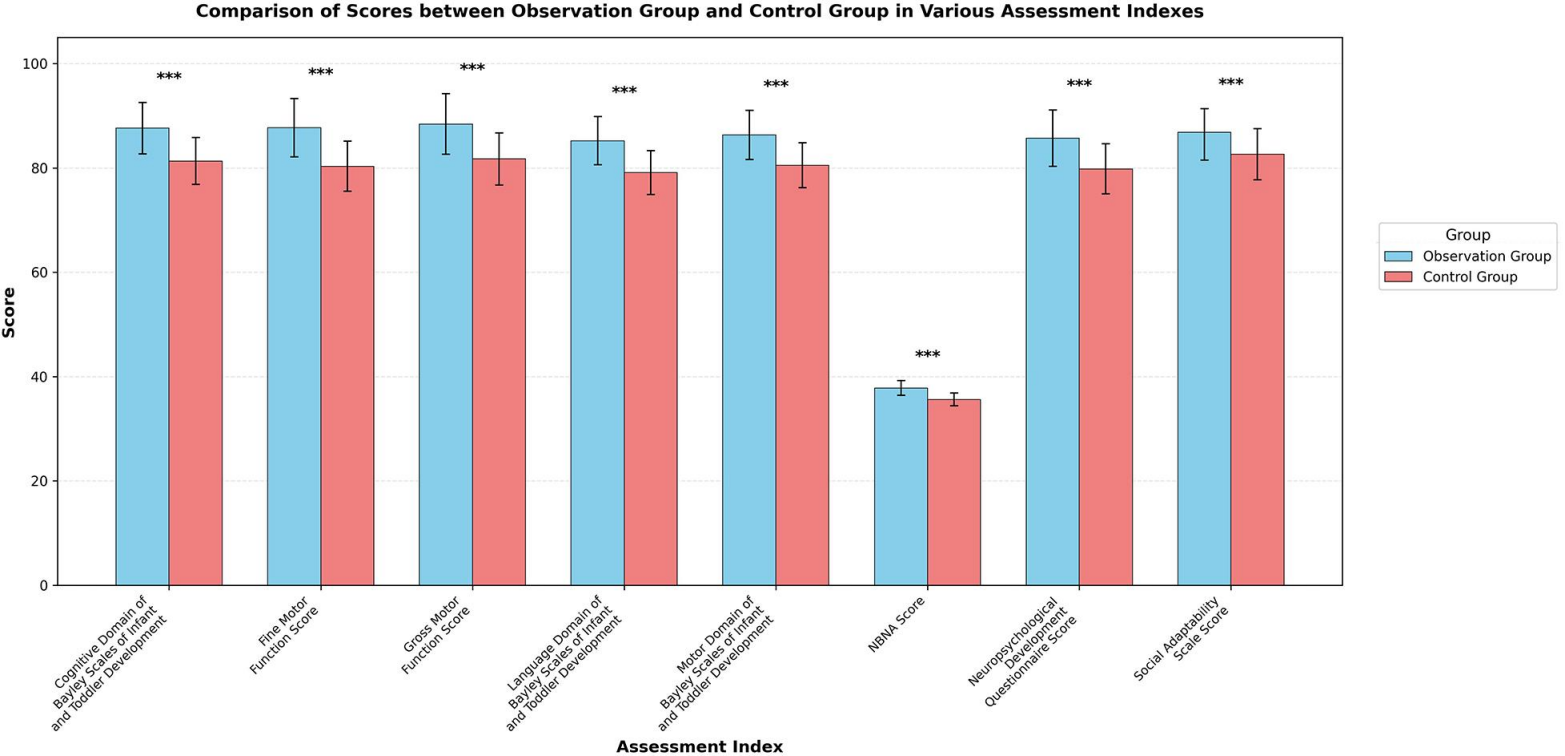
Comparison of neurodevelopmental assessment indicators between the two groups after intervention.

After the 6-month intervention, the scores of the Bayley Scales of Infant and Toddler Development in the observation group were significantly higher than those of the control group across all domains. In the cognitive domain, the observation group scored 87.6 ± 4.9 points compared with 81.3 ± 4.5 points in the control group (*t* = 8.867, *P* < 0.001, Cohen's d = 1.34, 95% CI: 1.02–1.66). In the language domain, the observation group scored 85.2 ± 4.6 points compared with 79.1 ± 4.2 points in the control group (*t* = 9.013, *P* < 0.001, Cohen's d = 1.39, 95% CI: 1.06–1.71). In the motor domain, the observation group scored 86.3 ± 4.7 points compared with 80.5 ± 4.3 points in the control group (*t* = 8.476, *P* < 0.001, Cohen's d = 1.29, 95% CI: 0.97–1.61). The NBNA score of the observation group (37.8 ± 1.4 points) was significantly higher than that of the control group (35.6 ± 1.2 points; *t* = 10.328, *P* < 0.001, Cohen's d = 1.69, 95% CI: 1.35–2.03). The gross motor function score of the observation group (88.4 ± 5.8 points) was significantly higher than that of the control group (81.7 ± 5.0 points; *t* = 7.772, *P* < 0.001, Cohen's d = 1.24, 95% CI: 0.92–1.55). The fine motor function score of the observation group (87.7 ± 5.6 points) was significantly higher than that of the control group (80.3 ± 4.8 points; *t* = 8.564, *P* < 0.001, Cohen's d = 1.42, 95% CI: 1.09–1.74). The Social Adaptability Scale score of the observation group (86.8 ± 5.3 points) was significantly higher than that of the control group (82.6 ± 4.9 points; *t* = 5.401, *P* < 0.001, Cohen's d = 0.82, 95% CI: 0.52–1.12). The Neuropsychological Development Questionnaire score of the observation group (85.7 ± 5.4 points) was significantly higher than that of the control group (79.8 ± 4.8 points; *t* = 7.637, *P* < 0.001, Cohen's d = 1.15, 95% CI: 0.84–1.47). All primary outcomes remained statistically significant after adjustment for multiple comparisons using the Benjamini-Hochberg procedure. These results are presented in Table 2 and Figure 2.

**Table 2:** Comparison of neurodevelopmental assessment indicators between the two groups after intervention.

Outcome measure	Observation (*n* = 93)	Control (*n* = 93)	t	*P*	Cohen's d
Bayley-III Cognitive	87.6 ± 4.9	81.3 ± 4.5	8.867	<0.001	1.34
Bayley-III Language	85.2 ± 4.6	79.1 ± 4.2	9.013	<0.001	1.39
Bayley-III Motor	86.3 ± 4.7	80.5 ± 4.3	8.476	<0.001	1.29
NBNA Score	37.8 ± 1.4	35.6 ± 1.2	10.328	<0.001	1.69
Gross Motor Function	88.4 ± 5.8	81.7 ± 5.0	7.772	<0.001	1.24
Fine Motor Function	87.7 ± 5.6	80.3 ± 4.8	8.564	<0.001	1.42
Social Adaptability Scale	86.8 ± 5.3	82.6 ± 4.9	5.401	<0.001	0.82
Neuropsychological Development	85.7 ± 5.4	79.8 ± 4.8	7.637	<0.001	1.15

Values expressed as mean ± SD. *P* values <0.001 are reported as “<0.001”. Cohen's d effect size interpretation: 0.2 = small, 0.5 = medium, 0.8 = large.

### 3.3 Comparison of daily behavior observation indicators

**Figure 3:**
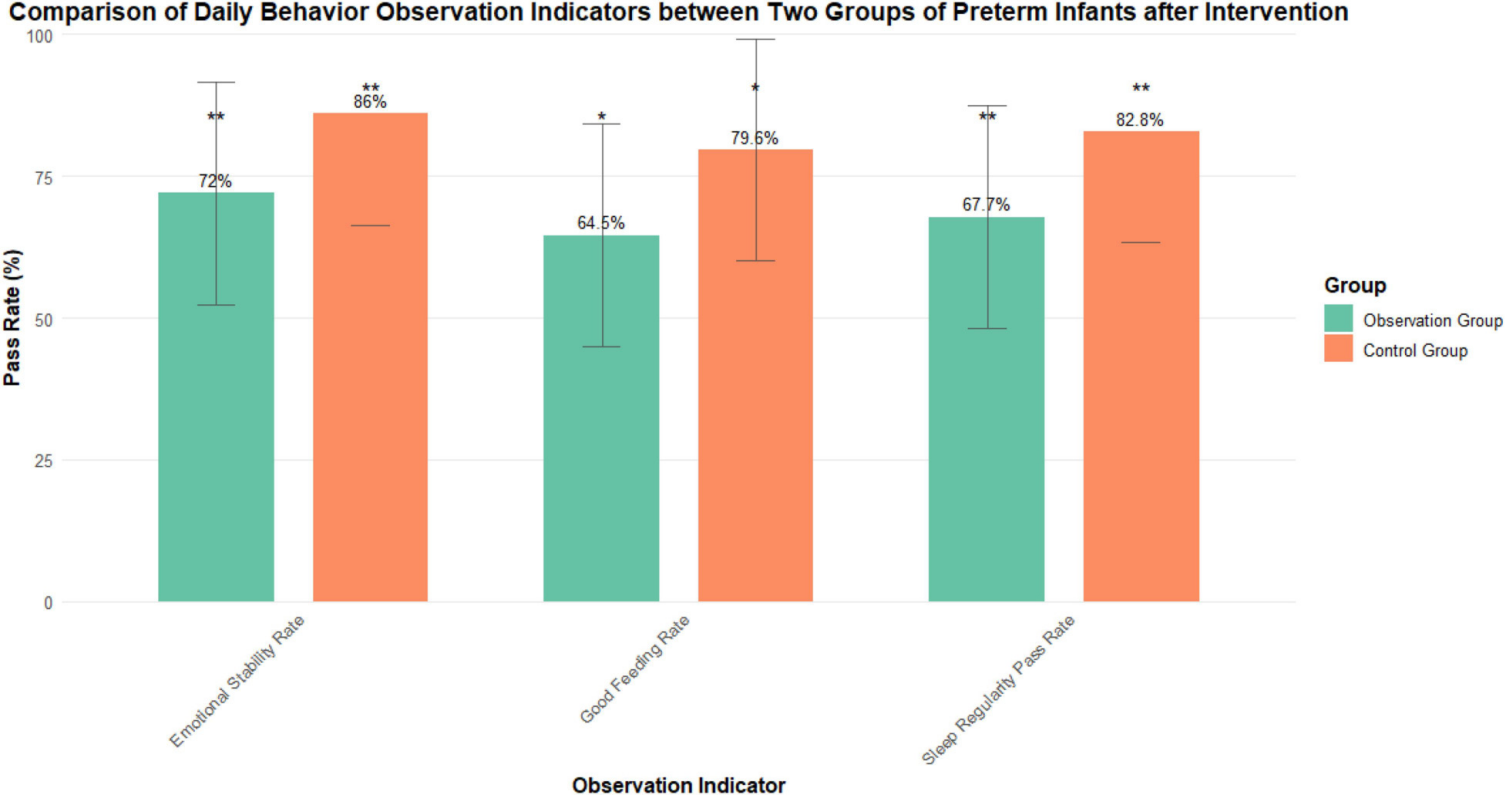
Comparison of daily behavioral observation indicators between the two groups after intervention.

The rate of meeting the standard of sleep regularity in the observation group was 82.8% (77/93), compared with 67.7% (63/93) in the control group (*χ*^2^ = 7.917, *P* = 0.005, Cramér's V = 0.21). The rate of good feeding in the observation group was 79.6% (74/93), compared with 64.5% (60/93) in the control group (*χ*^2^ = 6.356, *P* = 0.012, Cramér's V = 0.18). The rate of emotional stability in the observation group was 86.0% (80/93), compared with 72.0% (67/93) in the control group (*χ*^2^ = 7.021, *P* = 0.008, Cramér's V = 0.19). These results are presented in Table 3 and Figure 3.

**Table 3:** Comparison of daily behavioral observation indicators between the two groups after intervention.

Observation index	Observation (*n* = 93)	Control (*n* = 93)	χ^2^	*P*	Cramér's V
Sleep regularity pass rate	77 (82.8%)	63 (67.7%)	7.917	0.005	0.21
Good feeding rate	74 (79.6%)	60 (64.5%)	6.356	0.012	0.18
Emotional stability rate	80 (86.0%)	67 (72.0%)	7.021	0.008	0.19

Values expressed as *n* (%). Cramér's V effect size interpretation: 0.1 = small, 0.3 = medium, 0.5 = large.

## 4 Discussion

This randomized controlled trial examined the influence of family care guidance for premature infants based on a telemedicine model on their neurodevelopmental assessment indicators. The results demonstrate that the outcomes of the observation group were significantly superior to those of the control group across multiple neurodevelopmental assessment indicators and daily behavioral observation indicators. These findings have important implications for the practice of family care for premature infants.

This study was specifically designed to address the methodological deficiencies identified in previous research on telemedicine interventions for premature infants. Prior studies have been limited by inadequate sample sizes, lack of proper randomization, inconsistent outcome measures, and short follow-up periods (22, 23). The present study addressed these limitations through several design features. First, we employed rigorous randomization using a computer-generated sequence with allocation concealment. Second, we determined sample size through *a priori* power analysis, ensuring adequate statistical power to detect clinically meaningful differences. Third, we utilized well-validated, standardized neurodevelopmental assessment instruments (Bayley-III, NBNA, PDMS-2) with established psychometric properties. Fourth, outcome assessors were blinded to group assignment, reducing the risk of assessment bias. Fifth, we employed a 6-month intervention period, which is longer than many previous studies. These methodological improvements substantially enhance the reliability and validity of our findings compared with earlier research.

From the perspective of neurodevelopmental assessment indicators, the scores of the observation group in the cognitive, language, and motor domains of the Bayley Scales were significantly higher than those of the control group, with large effect sizes (Cohen's d ranging from 1.29 to 1.39). These results are consistent with findings from prior studies examining telemedicine-based interventions in premature infant populations (24, 30). The effect of maternal speech on neural development in premature infants has been demonstrated in previous research, and our intervention incorporated guidance on parent-infant verbal interaction (31). The telemedicine model, through regular remote video training, systematically imparted knowledge about the neurodevelopment of premature infants and early family intervention methods to parents. In the cognitive domain, parents learned how to promote sensory stimulation and cognitive development of premature infants through simple games and interactions. In the language domain, parents were guided to create a favorable environment for language development by communicating more with their infants and telling stories. Meanwhile, the real-time online consultation and personalized knowledge push functions ensured that parents could promptly obtain professional advice when encountering problems, continuously optimizing family care strategies and thereby effectively promoting the all-round development of premature infants (32).

In terms of the NBNA score, the observation group also performed well, with the largest effect size observed among all outcomes (Cohen's d = 1.69). The NBNA score reflects the neurobehavioral development level of premature infants, including multiple aspects such as behavioral ability, muscle tone, and primitive reflexes (26). Under the telemedicine model, medical staff could promptly correct improper operations of parents in daily care, such as correct holding postures and tactile stimulation techniques. These operations are helpful in improving the muscle tone and neural reflexes of premature infants, thereby enhancing the NBNA score (33). Research has demonstrated that tactile experience during preterm infant feeding has positive effects on clinical outcomes, which supports the mechanisms underlying our intervention effects (34). In addition, personalized knowledge push provided targeted nursing suggestions based on the individual differences of premature infants. For example, for premature infants with abnormal muscle tone, specific rehabilitation training methods were pushed to further promote the normal development of their neurobehavior (35).

The assessment results of gross motor and fine motor functions indicated that the scores in the observation group were significantly higher than those in the control group, with large effect sizes (Cohen's d = 1.24 and 1.42, respectively). Premature infants require scientific intervention measures to promote the development of their motor functions due to delayed early motor development (36). Hospital-home intervention protocols for motor development in premature infants have shown promising results in previous research (7). The early family intervention methods provided by telemedicine, such as passive motor training and prone head raising exercises, guided parents to conduct systematic motor training for premature infants at home. Meanwhile, through real-time online consultation, parents could promptly provide feedback on the problems encountered during the training process, and medical staff offered adjustment suggestions to ensure the effectiveness and safety of the training (37). Long-term adherence to these scientific exercise trainings enabled the premature infants in the observation group to achieve better development in both gross motor skills and fine motor skills.

The scores of the Social Adaptability Scale and the Neuropsychological Development Questionnaire showed that the observation group was superior to the control group in terms of social adaptability and neuropsychological development. This result reflects the positive impact of the telemedicine model on the psychological and social development of premature infants (38). Through remote video training, parents learned how to cultivate good emotions and social interaction skills in premature infants, such as enhancing emotional connections through parent-child interactive games, creating opportunities for premature infants to interact with the outside world, and promoting the development of their social adaptability. Parent-infant interaction during the first year of life has been shown to be particularly important for infants at high risk for developmental delay (37). Personalized knowledge push also provided information about the psychological development characteristics and coping strategies of premature infants, helping parents pay attention to the psychological needs of premature infants and create a favorable psychological development environment for them (39).

In terms of the indicators of daily behavior observation, the rates of meeting the standards of sleep regularity, good feeding, and emotional stability in the observation group were significantly higher than those in the control group, with small-to-medium effect sizes (Cramér's V ranging from 0.18 to 0.21). Good sleep, feeding, and emotional state are important guarantees for the healthy growth of premature infants (40). The telemedicine model provided parents with scientific guidance on sleep, feeding, and emotional management. For example, parents were guided to establish a regular sleep schedule and improve the feeding quality of premature infants by adjusting the feeding methods and times (41). When premature infants had emotional problems, parents could obtain professional soothing suggestions through real-time online consultation to stabilize the emotions of premature infants in a timely manner (42). These scientific nursing guidelines enabled the premature infants in the observation group to maintain better conditions in their daily lives, providing favorable conditions for neural development.

The 0% attrition rate observed in this study merits discussion, as retention rates in longitudinal studies of premature infants typically range from 70% to 90%. Several factors likely contributed to this exceptional retention. First, the telemedicine intervention itself maintained continuous engagement with families in the observation group, with monthly video sessions and weekly knowledge push creating regular contact points. Second, parents of premature infants are often highly motivated to ensure optimal developmental outcomes for their children and may therefore be more committed to research participation. Third, the convenience of the telemedicine format eliminated transportation barriers that often contribute to attrition. Fourth, control group families remained engaged through regular outpatient follow-up visits that were part of standard care. Fifth, assessment at 6 months rather than at later time points minimized the opportunity for attrition. While this exceptional retention is a strength of the study, it may also limit generalizability to settings where engagement is less intensive.

Regarding the feasibility of this intervention becoming the standard of care, several factors support its potential for widespread implementation. The intervention utilized existing telemedicine infrastructure and freely available communication platforms (WeChat), minimizing additional resource requirements. Medical personnel training for telemedicine delivery was straightforward and could be integrated into existing professional development programs. Family acceptance was high, as evidenced by the excellent adherence rates (95.0% attendance at video sessions). The intervention was delivered without dedicated funding, demonstrating feasibility within existing resource constraints. However, successful implementation at scale would require institutional commitment to telemedicine infrastructure, training programs for healthcare providers, and protocols for quality assurance. Additionally, families must have access to smartphones and reliable internet connectivity, which may remain barriers in some populations.

This study has several limitations that should be considered when interpreting the results. First, the study was conducted at a single tertiary hospital in an urban setting, and the situation of premature infants in different regions and hospitals may vary. Future studies should expand the sample range and include multi-center research subjects to enhance the generalizability of the research results. Second, the intervention period was 6 months, which, while longer than many previous studies, may not capture the full long-term neurodevelopmental impact on premature infants. Further follow-up studies are needed to understand the sustained effects of the telemedicine model on neurodevelopment. Third, while randomization was employed, there was no attempt to match the two groups of infants according to baseline neurodevelopmental status beyond the pre-discharge NBNA assessment. Although no baseline differences were detected, the absence of comprehensive baseline developmental testing limits confidence in attributing post-intervention differences solely to the intervention. Fourth, without baseline developmental scores on all outcome measures, between-group comparisons cannot include confidence intervals for change scores, preventing inspection for overlap in individual-level change. Fifth, while we reported intervention dose (number of sessions attended and consultations), we did not systematically control for the number of interactions the experimental group had with professionals, which may represent a confounding factor independent of the specific telemedicine content. Sixth, the control group did not receive an attention-control intervention, leaving open the possibility that some benefits arose from increased contact with healthcare providers rather than the specific content of the telemedicine intervention. Seventh, this study did not delve deeply into possible influencing factors during the implementation of the telemedicine model, such as the acceptance level of parents and network technology issues. These factors may affect the intervention effect and require further attention and analysis in subsequent studies.

## 5 Conclusion

In conclusion, family care guidance for premature infants based on a telemedicine model has a significant effect on improving the neurodevelopmental assessment indicators and daily behavioral performance of premature infants. By addressing methodological limitations of previous research through rigorous randomization, blinded assessment, validated outcome measures, and adequate sample size, this study provides robust evidence for an efficient and convenient new approach for family care of premature infants. Although certain limitations remain, the application value of this model is worthy of recognition. In the future, it should be further optimized through multi-center trials with longer follow-up periods, and the feasibility of implementation as standard of care should be formally evaluated to better serve the healthy growth of premature infants.

## Data Availability

The original contributions presented in the study are included in the article/Supplementary Material, further inquiries can be directed to the corresponding author.
